# Symptoms and Immune Markers in *Plasmodium*/Dengue Virus Co-infection Compared with Mono-infection with Either in Peru

**DOI:** 10.1371/journal.pntd.0004646

**Published:** 2016-04-29

**Authors:** Eric S. Halsey, G. Christian Baldeviano, Kimberly A. Edgel, Stalin Vilcarromero, Moises Sihuincha, Andres G. Lescano

**Affiliations:** 1 Virology Department, U.S. Naval Medical Research Unit No. 6, Lima and Iquitos, Peru; 2 The President’s Malaria Initiative, Centers for Disease Control and Prevention, Atlanta, Georgia, United States of America; 3 Malaria Branch, Division of Parasitic Diseases and Malaria, Centers for Disease Control and Prevention, Atlanta, Georgia, United States of America; 4 Parasitology Department, U.S. Naval Medical Research Unit No. 6, Lima, Peru; 5 Infectious Diseases Department, Hospital de Apoyo, DISA-Loreto, Iquitos, Peru; 6 School of Public Health and Administration, Universidad Peruana Cayetano Heredia, Lima, Peru; Pediatric Dengue Vaccine Initiative, UNITED STATES

## Abstract

**Background:**

Malaria and dengue are two of the most common vector-borne diseases in the world, but co-infection is rarely described, and immunologic comparisons of co-infection with mono-infection are lacking.

**Methodology and Principal Findings:**

*We* collected symptom histories and blood specimens from subjects in a febrile illness surveillance study conducted in Iquitos and Puerto Maldonado, Peru, between 2002–2011. Nineteen symptoms and 18 immune markers at presentation were compared among those with co-infection with *Plasmodium*/dengue virus (DENV), *Plasmodium* mono-infection, and DENV mono-infection. Seventeen subjects were identified as having *Plasmodium*/DENV co-infection and were retrospectively matched with 51 DENV mono-infected and 44 *Plasmodium* mono-infected subjects. Those with *Plasmodium* mono-infection had higher levels of IL-4, IL-6, IL-10, IL-12, IL-13, IL-17A, IFN-γ, and MIP1-α/CCL3 compared with DENV mono-infection or co-infection; those with *Plasmodium* mono-infection had more cough than those with DENV mono-infection. Subjects with DENV mono-infection had higher levels of TGF-β1 and more myalgia than those with *Plasmodium* mono-infection. No symptom was more common and no immune marker level was higher in the co-infected group, which had similar findings to the DENV mono-infected subjects.

**Conclusions/Significance:**

Compared with mono-infection with either pathogen, *Plasmodium*/DENV co-infection was not associated with worse disease and resembled DENV mono-infection in both symptom frequency and immune marker level.

## Author Summary

Dengue and malaria are two of the most important diseases spread by mosquitoes. Clinical manifestations of both febrile diseases overlap considerably, and either can be fatal. In addition, they are co-endemic in many places throughout the world. Despite this, only a handful of reports of co-infection with dengue virus and *Plasmodium* species are reported in the literature. Through our febrile surveillance program in the Peruvian Amazon, we were able to retrospectively identify 17 cases of co-infection with dengue virus and *Plasmodium*. Our study aimed to assess whether co-infection was associated with more symptoms or a different immune response compared with mono-infection alone. To answer this question, we utilized data and blood specimens collected during the acute presentation of these 17 subjects and compared them to a matched group of subjects with either dengue virus mono-infection or *Plasmodium* mono-infection. Our findings indicate co-infection with both pathogens was not associated with more symptoms and the immune profile of co-infection resembled dengue virus mono-infection more than *Plasmodium* mono-infection.

## Introduction

Malaria and dengue fever are two of the most important mosquito-borne infections affecting humans. Annually, approximately 584,000 and 12,000 people die and another 198 million and 96 million are estimated to fall ill from malaria and dengue, respectively [1, 2]. Although *Anopheles*, the mosquito vector of malaria, and *Aedes*, the mosquito vector of dengue virus (DENV), occupy different ecological niches, considerable geographical overlap in disease risk exists for the 3.3 and 4.0 billion people who live in an area endemic for malaria and dengue, respectively [2, 3]. The two diseases also share many common clinical features—including fever, headache, body aches, and fatigue—making one disease easily mistaken for the other.

Limited availability of diagnostics may be a reason only a handful of reports describe infection with *Plasmodium* species (which will be abbreviated *Plasmodium*) and DENV each year (Table 1) [4–34]. The first reports of co-infection occurred in 2005 [4, 6] and most subsequent reports describe only individual patients and rely on serological methods for diagnosing DENV infection. The majority originate from India and nearby Pakistan, although West Africa [6], French Guiana [11, 17], Brazil [19, 23, 27], Malaysia [25], Bangladesh [33, 34], East Timor [9], Thailand [26], and Indonesia [24] have also been the origin of case reports.

**Table 1:** Case reports and case series of co-infection with *Plasmodium* and dengue virus.

Reference	Year	Country	# of subjects with co-infection	*Plasmodium* species	Method of Malaria Diagnosis	DENV serotype	Method of Dengue Diagnosis	Death	Observations
Arya	2005	India	2	Pv	microscopy	NR	IgM	No	
Charrel	2005	Guinea, Senegal, and Sierra Leone	1	Pf	microscopy	3	culture, IgM	NR	
Bhalla	2006	India	1	Pf	microscopy	NR	IgM	No	
Ali	2006	Pakistan	9	Pf = 1Pv = 8	microscopy	NR	IgM	Yes	One death occurred in the only Pf-infected subject
Deresinski	2006	India	1	Pv	microscopy	NR	IgM	No	
Ward	2006	East Timor	1	Pf	microscopy (RDT negative)	NR	IgM	Yes	
Thangaratham	2006	India	1	Pv	microscopy	2	culture, IgM	No	
Kaushik	2007	India	1	Pf & Pv	microscopy, RDT	NR	IgM	No	Three pathogens: DENV, Pf, and Pv
Abassi	2009	Pakistan	26	Pf = 1Pv = 25	microscopy	NR	IgM	No	Compared co-infections with DENV mono-infection; more anemia and thrombocytopenia in co-infection; more leukopenia in DENV mono-infection
Chaudhry	2009	India	1	Pv	microscopy	NR	IgM	No	Four pathogens: DENV, hepatitis E virus, *Leptospira* sp, and Pv
Chander	2009	India	1	Pf	microscopy	NR	IgM	No	
Carme	2009	French Guiana	17	Pf = 3Pv = 14	microscopy	1,3	culture, IgM, PCR	No	
Santana	2010	Brazil	2	Pv	microscopy	2	culture, PCR	NR	
Magalhães	2012	Brazil	11	Pv	microscopy, PCR	2,3,4	NS1, PCR	No	Three pathogens: DENV-3, DENV-4, and Pv
Epelboin	2012	French Guiana	104	Pf = 21, Pv = 80, Pf & Pv = 3	microscopy	1,2,3	IgA, IgM, NS1, PCR	NR	Compared co-infection with mono-infections; more severe and more hematologic abnormalities in co-infection
Yong	2012	Indonesia	1	Pf	microscopy	NR	IgM	No	Experienced rhabdomyolysis and acute renal failure requiring dialysis; patient visited Indonesia but was evaluated in Singapore
Mohapatra	2012	India	27	Pf = 24, Pv = 2, Pf & Pv = 1	microscopy	NR	IgM, NS1	No	Co-infection much more like dengue than malaria
Hati	2012	India	46	Pf = 18, Pv = 28	microscopy	NR	IgM	No	Co-infection more common during certain times of the year
Malhotra	2012	India	1	Pv	microscopy, RDT	NR	IgM, NS1	No	
Faruque	2012	Bangladesh	1	Pv	microscopy, RDT	NR	IgM	NR	
Mushtaq	2013	India	1	Pf & Pv	microscopy, RDT	NR	IgM	No	Three pathogens: DENV, Pf, and Pv
Yong	2013	Malaysia	1	Pv	microscopy	NR	IgM	No	Three pathogens: DENV, *Leptospira* sp, and Pv
Alam	2013	India	1	Pf	microscopy, RDT	NR	NS1 (IgM negative)	No	Cerebral malaria
Assir	2013	Pakistan	17	Pf = 3, Pv = 14	microscopy	NR	IgM, NS1, PCR	No	Co-infection similar to mono-infection for most clinical features; more jaundice in mono-infection with *Plasmodium* or DENV than with co-infection
Pande	2013	India	1	Pf & Pv	microscopy, RDT	NR	IgM, NS1	No	Three pathogens: DENV, Pf, and PvPregnant
Magalhães	2014	Brazil	44	Pv	microscopy, PCR	2,4	IgM, NS1, PCR	No	Co-infection had more jaundice, hepatomegaly, and severe disease than dengue; co-infection had more bleeding than malaria
Satyawali	2014	India	1	Pf	microscopy, RDT	NR	IgM, NS1	No	Three pathogens: DENV, Pf, *Wuchereria bancrofti*
Issaranggoon	2014	Thailand	1	Pf	microscopy	NR	IgM, NS1	No	
Kumar	2014	India	1	Pv	microscopy, RDT	NR	IgM, NS1	No	Three pathogens: DENV, *Orientia tsutsugamushi*, and Pv
Bhagat	2014	India	3	Pv	microscopy, RDT	NR	IgM, NS1	No	
Swoboda	2014	Bangladesh	19	NR	microscopy, RDT	NR	IgM	NR	One also had typhoid fever and 13 also had leptospirosis

DENV: dengue virus; Pf: *Plasmodium falciparum*; Pv: *Plasmodium vivax*; NR: not reported; ICT: immunochromatographic test; NS1: dengue antigen NS1; PCR: polymerase chain reaction; RDT: rapid diagnostic test

The immune responses against DENV and *Plasmodium* parasites are unique and complex, although similarities between the two responses exist [35, 36]. Both pathogens elicit a robust antibody response with the ability to neutralize receptor-mediated invasion of host cells [36, 37]. In addition, DENV and *Plasmodium* possess pattern recognition molecules which induce the release of inflammatory molecules, including interleukin (IL)-1β, IL-6, IL-8 and tumor necrosis factor alpha (TNF-α) [36, 37]. Because both DENV and *Plasmodium* are intracellular pathogens, the induction of Th1 cytokines (e.g., IL-2, IL-12 and gamma interferon [IFN]-γ) is critical for the activation of macrophages, cytotoxic T cells, and other cellular-mediated mechanisms involved in controlling replication of the pathogens. In contrast, the up-regulation of Th2 cytokines (e.g., IL-4, IL-5, and IL-13) and regulatory cytokines (e.g., IL-10 and transforming growth factor [TGF]-β1) is associated with immune escape and disease progression following DENV or *Plasmodium* infection [38].

Despite the need for the activation of inflammatory responses during the development of protective immunity, activation of these pathways may also result in tissue damage and severe disease [35, 36, 39]. Several of the pro-inflammatory cytokines induced by DENV or *Plasmodium*, such as IL-6, TNF-α, and IL-1β, are important mediators of acute phase proteins, as well as mechanisms of endothelial cell activation and secretion of chemokines. These molecules contribute to increased vascular permeability during severe dengue or are associated with sequestration of parasitized red blood cells during severe malaria, particularly cerebral malaria. Conversely, the induction of regulatory cytokines such as IL-10 has been involved in protection from severe disease in both dengue and malaria. Thus, the modulation of the inflammatory response following DENV or *Plasmodium* infection likely influences the onset of symptoms and disease outcome. Because DENV and *Plasmodium* both induce a type-1 immune response, co-infection theoretically could result in either enhanced pathogen clearance or increased immunopathogenesis. Given the overlapping endemicities of these two infections, it is important to understand the immune interactions that occur during co-infection, as they may lead to improved management of such cases or the identification of biomarkers to better identify co-infected cases.

To characterize symptoms and immune marker secretion profile associated with *Plasmodium*/DENV co-infection, we conducted an exploratory, retrospective comparative study in which we identified 17 cases with *Plasmodium*/DENV co-infection and matched *Plasmodium* and DENV mono-infected controls. These cases were identified through a febrile surveillance cohort conducted from 2002 to 2011 in Iquitos and Puerto Maldonado in the Peruvian Amazon Basin.

## Methods

### Ethics statement

This study utilized institutional review board (IRB)-approved protocols 2000.0006 (U.S. Naval Medical Research Center; Silver Spring, MD), 2010.0010 (U.S. Naval Medical Research Unit No. 6 (NAMRU-6); Lima, Peru), and 2012.0002 (NAMRU-6), in compliance with all U.S. Federal regulations governing the protection of human subjects. Protocols 2000.0006 and 2010.0010 were also reviewed by the Peruvian Ministry of Health. After being informed of the study, written consent was obtained from all participating adults or a parent or legal guardian of subjects 5–17 years old. Assent was also obtained from subjects between ages 8–17 years old. Protocol 2012.0002 covered immune marker testing of the specimens collected in the two aforementioned protocols.

### Patient recruitment

From January 2002 to March 2011, subjects with an undifferentiated febrile illness were recruited at outpatient clinics and screened for the presence of an arboviral infection, as described previously [40]. This population is almost entirely formed by Peruvian citizens of mestizo origin. For this project, we only included local residents eligible for healthcare in the Peruvian cities of Iquitos and Puerto Maldonado because the Peruvian Ministry of Health often screened febrile subjects for malaria in addition to our arboviral assays to detect infection with DENV. Because the Ministry of Health’s malaria screening was only incorporated into the febrile surveillance during certain time periods, samples—both mono-infected and co-infected—were only selected from these time periods. Inclusion criteria for febrile surveillance were: 1) oral/tympanic temperature > 38.0°C (or axillary > 37.5°C) or report of fever starting within the previous 5 days, 2) no obvious source of infection such as cellulitis, dental abscess, or urinary tract infection, and 3) age ≥ 5 years. Subjects infected with two serotypes of DENV (e.g., both DENV-1 and DENV-3) or two species of *Plasmodium* (e.g., both *Plasmodium vivax* and *Plasmodium falciparum*) were excluded from the analysis.

### Collection of symptom data

Each site used a standardized questionnaire which collected the demographic and symptom information assessed in this study. In addition, all participating healthcare personnel received standardized training in data collection. We assessed 19 symptoms in our analysis: abdominal pain, nausea, vomiting, diarrhea, rhinorrhea, cough, expectoration, epistaxis, bleeding gums, hematuria, hematochezia, prostration, asthenia, chills, malaise, arthralgia, myalgia, eye pain, and headache.

### Laboratory determination of DENV and *Plasmodium* infection

Both a 10 ml serum and a 2 ml whole blood sample were collected from febrile subjects. Microscopy was the primary method of identifying *Plasmodium* and culture was the primary method of identifying DENV, but DENV and *Plasmodium*-specific polymerase chain reaction (PCR) were used to augment these modalities when sufficient sample was available, as will be discussed later in this paragraph. A portion of the whole blood sample was used for malaria microscopy, performed at the site of collection by the Peruvian Ministry of Health using thick and thin smears according to national guidelines [41]. The remainder of the whole blood sample and the serum sample were stored at -80°C until transfer to NAMRU-6 on dry ice. At NAMRU-6, if sufficient quantity of whole blood existed, the infecting *Plasmodium* species was confirmed by PCR [42] on samples deemed positive by smear; *Plasmodium* PCR was also used to further rule out the presence of *Plasmodium* in smear-negative, DENV-positive specimens. To assess for the presence of DENV, serum was inoculated on C6/36 and Vero cell cultures, as previously described [40]. After ten days or upon appearance of cytopathic effect, immunofluorescent antibodies to a wide range of endemic arboviruses, including the four DENV serotypes, were added. PCR for DENV was also performed on a subset of samples, as previously described [40]. Primary versus secondary DENV infection was determined using a ratio of enzyme linked immunosorbent assay (ELISA) titers for IgM and IgG, similar to an approach used by others [43].

### Classification/selection of co-infected and mono-infected cases

A subject was determined to be mono-infected with *Plasmodium* if they had a positive malaria smear and a negative viral culture and ELISA IgM for DENV. A subject was determined to be mono-infected with DENV if they had a positive culture or PCR for DENV and negative malaria microscopy (and PCR, if sufficient quantity of whole blood existed). A subject was determined to be co-infected if both a DENV assay (culture or PCR) and malaria microscopy were positive.

First, we identified all co-infections from the two study sites in the project database. We then selected three DENV mono-infected subjects and two to three *Plasmodium* mono-infected subjects for each co-infected subject (two co-infected subjects did not have *Plasmodium* mono-infected controls) to compare symptom frequency and levels of immune markers. All mono-infected control subjects were individually matched to a co-infected case by recruitment site, time of enrollment (±3 months), gender, and DENV serotype. Pregnant subjects were excluded from analysis due to the difficulty in finding adequately matched control subjects.

### Measurement of immune markers

Serum immune marker levels were measured using the xMAP platform (Luminex; Austin, TX), following the manufacturer’s recommendations. A 10-point calibration curve was used instead of an 8-point calibration curve to improve performance at the lower range of detection. The reactions were detected using the Bio-plex 100 (Bio-Rad Laboratories; Hercules, CA) system, and cytokine, chemokine, and growth factor concentrations were calculated using Bio-plex Manager Software (Bio-Rad Laboratories). The limit of detection (LOD) was defined as the lowest calibrator value at which the coefficient of variance of concentration was less than 25% and recovery of calibrator was within 20% of the expected value. All values below the LOD were considered undetectable and assigned a value equal to half the plate-specific LOD. TGF-β1 was detected using a commercially available ELISA assay (R&D Systems; Minneapolis, MN). The 18 immune markers tested belonged to the following categories: inflammatory acute response cytokines (IL-1β, IL-6, IL-8, TNF-α); chemokines and growth factors (IL-2, IL-7, granulocyte colony stimulating factor [G-CSF], monocyte chemoattractant protein [MCP]-1/CCL2, macrophage inflammatory protein [MIP]-1α/CCL3); inflammatory Th1 cytokines (IL-12, IFN-γ); inflammatory Th2 cytokines (IL-4, IL-5, IL-13); inflammatory Th17 cytokines (IL-17A, granulocyte macrophage colony stimulating factor [GM-CSF]); and regulatory cytokines (IL-10, TGF-β1). For IL-12, the assay detected the p70 protein; therefore, our assay was specific for IL-12 and did not cross-react with IL-23. All samples were tested in the same batch under the same conditions. Immune markers with values undetectable in more than 75% of the samples were not included in the analysis.

### Statistical analysis

Symptom frequencies among the three groups were compared using conditional logistic regression, using a separate regression to compare the frequency of each individual symptom between co- and mono-infections. The concentrations of immune markers were compared among the three groups using the Kruskal-Wallis test, a non-parametric method that is robust and less affected by outliers. Log-transformed cytokine concentrations were compared between the three groups using random effects linear regression, again with a separate regression for each immune marker. Age and number of days with fever, and the influence of other covariates not matched for in the study design were adjusted in the regression analyses. The sample size available had 80% power to detect a difference of 0.8 standard deviations in immune marker levels between co- and mono-infected individuals.

## Results

Seventeen co-infected subjects (14 *P*. *vivax* and 3 *P*. *falciparum*; 2 DENV-1 and 15 DENV-3) were identified and included in the study (Table 2). *Plasmodium* infection was initially identified with microscopy in all subjects and further confirmed with PCR in five (adequate sample was not available to perform confirmatory PCR on the others). DENV infection was established with culture in 15 subjects and with PCR in two subjects. Using the aforementioned matching criteria, 51 DENV mono-infected subjects and 44 *Plasmodium* mono-infected subjects were selected for comparison. The age distribution was similar among the three groups. The frequencies of the DENV serotypes were similar between the co-infected and DENV mono-infected subjects and the frequencies of the *Plasmodium* species were similar between the co-infected and *Plasmodium* mono-infected subjects. The average number of days with symptoms was lower in DENV mono-infected subjects compared to *Plasmodium* mono-infected or co-infected subjects (p<0.05; Table 2).

**Table 2:** Characteristics of the co-infected, dengue virus mono-infected, and *Plasmodium* mono-infected groups.

		Co-infected (n = 17)	DENV(n = 51)	*Plasmodium*(n = 44)	p value**
**Items not matched**	** **				
	**Mean age (SD)**	29.0 (17.3)	27.8 (13.1)	29.6 (16.1)	0.845
	**Days of symptoms at presentation (SD)**	3.1 (1.7)	2.1 (1.3)	3.1 (1.8)	0.006
	**DENV infection**				0.309
	Primary	8 (47%)	17 (33%)	N/A	
	Secondary	9 (53%)	34 (67%)	N/A	
	**Malaria species**				0.638
	*P*. *vivax*	14 (82%)	N/A	36 (82%)	
	*P*. *falciparum*	3 (18%)	N/A	8 (18%)	
Items matched*	** **				
	**Gender**				
	male	12 (71%)	36 (71%)	29 (66%)	
	female	5 (29%)	15 (29%)	15 (34%)	
	**Location**				
	Iquitos	16 (94%)	48 (94%)	44 (100%)	
	Puerto Maldonado	1 (6%)	3 (6%)	0	
	**DENV serotype**				
	DENV-1	2 (12%)	6 (12%)	N/A	
	DENV-3	15 (88%)	45 (88%)	N/A	

*Subjects were also matched by date of enrollment (±3 months)

**p values determined using chi square, Fisher's exact, and ANOVA; DENV: dengue virus; SD: standard deviation

Only two symptoms were significantly different (p<0.05) upon comparison among the three groups: 1) more frequent myalgia in DENV mono-infected vs *Plasmodium* mono-infected subjects, and 2) more frequent cough in *Plasmodium* mono-infected vs DENV mono-infected subjects (Table 3). These differences remained significant after adjusting for age and days since symptoms onset. No symptoms were significantly more frequent in co-infection cases. Only two subjects reported epistaxis, one in the co-infected group and one in the DENV mono-infected group. No subject reported any of the other hemorrhagic manifestations—gum bleeding, hematochezia, or hematuria.

**Table 3:** Symptom frequency in subjects with co-infection, dengue virus (DENV) mono-infection, and *Plasmodium* mono-infection.

Symptoms	Co-infection n/total (%)	DENV n/total (%)	*Plasmodium* n/total (%)	p value*
Abdominal pain	8/17 (47%)	15/51 (29%)	21/44 (48%)	0.1033
Epistaxis***	1/17 (7%)	1/51 (2%)	0/44 (0%)	0.282
Prostration	2/15 (13%)	2/51 (4%)	3/41 (7%)	0.5625
Asthenia	9/16 (56%)	23/51 (45%)	18/44 (41%)	0.3873
Chills	15/17 (88%)	48/51 (94%)	41/44 (93%)	0.7081
Malaise	15/16 (94%)	50/51 (98%)	41/41 (100%)	0.4286
Pallor	5/16 (31%)	11/51 (22%)	18/44 (41%)	0.2133
Rash	2/16 (13%)	2/51 (4%)	0/41 (0%)	0.0445
Arthralgia	14/16 (88%)	48/51 (94%)	39/44 (87%)	0.4844
Myalgia	13/16 (81%)	45/51 (88%)	30/44 (68%)	0.031**
Diarrhea	4/17 (24%)	7/51 (14%)	7/44 (16%)	0.6376
Nausea	13/17 (76%)	36/51 (71%)	28/44 (64%)	0.4135
Vomiting	8/17 (47%)	23/51 (45%)	16/44 (36%)	0.2949
Rhinorrhea	2/16 (13%)	4/50 (8%)	6/44 (14%)	0.8348
Cough	5/16 (31%)	5/51 (10%)	15/44 (34%)	0.010**
Expectoration	1/16 (6%)	2/51 (4%)	3/44 (7%)	0.8648
Headache	16/16 (100%)	51/51 (100%)	42/44 (95%)	0.1837
Eye pain	10/15 (67%)	40/51 (78%)	28/41 (68%)	0.455

* Conditional logistic regression, matched analyses

** Dengue virus mono-infection different from *Plasmodium* mono-infection

*** No recorded cases of gum bleeding, hematochezia, or hematuria

**Fig 1:**
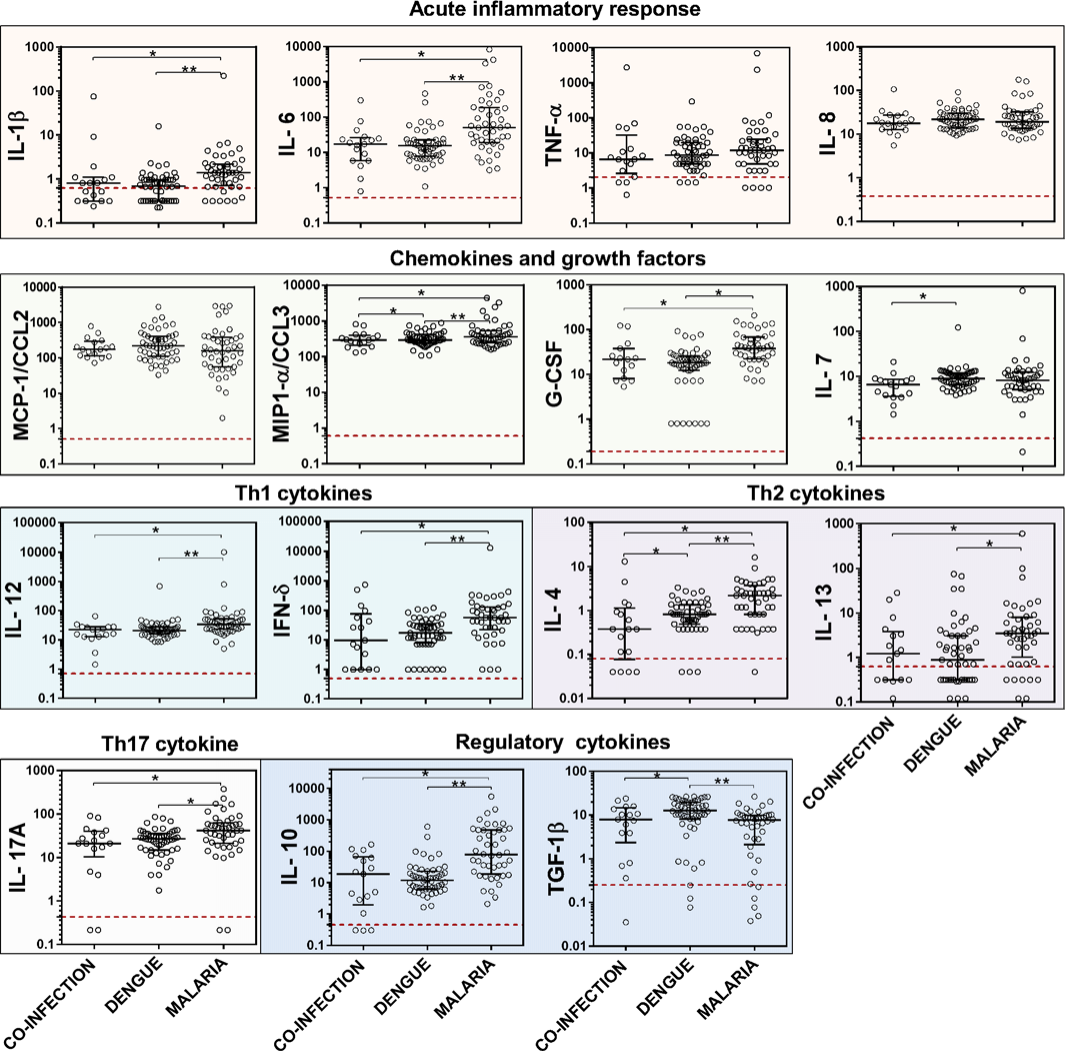
Serum levels of immune markers among patients with *Plasmodium*/DENV co-infection, DENV mono-infection, and *Plasmodium* mono-infection. Serum levels were analyzed by 17-plex cytokine array (Biorad Laboratories) for all immune markers except TGF-β1, which was measured by an R&D Systems ELISA kit. Three immune markers were excluded (GM-CSF, IL-5, and IL-2) from this analysis because more than 25% of the values fell below the limit of detection of the assays. Horizontal bars represent group median and inter-quartile range. Red dotted lines show lower limit of detection for each immune marker. Units on the y-axis are log 10 transformed scales of analyte concentration in ng/mL for all immune markers except for TGF-β1, which is represented in μg/mL. * p<0.05 for group comparison by the Kruskal-Wallis test followed by post-hoc Dunn’s pair-wise comparison.

To examine the immune profile of individuals with co-infection, we compared the concentration of 15 immune markers (IL-1β, IL-6, IL-8, TNF-α, IL-7, G-CSF, MCP-1/CCL2, MIP-1-α/CCL3, IL-12, IFN-γ, IL-13, IL-17A, GM-CSF, IL-10, and TGF-β1) among the three groups using Kruskal-Wallis ANOVA. Three other immune markers (IL-2, IL-5, and GM-CSF) were excluded from the analysis given their high frequency of measurements falling below the LOD. The *Plasmodium* mono-infected group had significantly (p<0.05) higher concentrations for the majority of the immune markers tested compared to the DENV mono-infected or the co-infected groups (Fig 1). The consistently higher levels in *Plasmodium* mono-infected subjects were not particular to a specific type of immune marker, as significant differences were found in the inflammatory acute response (IL-1β, IL-6), chemokine and growth factor (G-CSF), Th1 (IL-12, IFN-γ), Th2 (IL-4), Th17 (IL-17A), and the regulatory cytokine IL-10. In contrast, the concentration of TGFβ1, a regulatory cytokine, was significantly higher in the DENV mono-infected group compared to either the *Plasmodium* mono-infected or co-infected groups. DENV mono-infected subjects and co-infected subjects had similar levels for all other immune markers except IL-4 and IL-7, which were both higher in the DENV mono-infected group compared to the co-infected group. IL-4 was the only immune marker with statistically significant differences across the three groups, with the *Plasmodium* mono-infected group having the highest and the co-infected group the lowest level (Fig 1). No immune marker was significantly higher in the co-infected group compared to either of the mono-infected groups.

To assess for the influence of other covariates which were not matched for in the study design, we fitted a random effects regression model for matched group analysis, adjusting for age and number of days with fever. This analysis confirmed the above results in that the *Plasmodium* mono-infected subjects had significantly higher serum concentrations of IL-4, IL-6, IL-10, IL-12, IL-13, IL-17A, MIP-1α/CCL3, and IFN-γ (p < 0.05) than either the co-infected or the DENV mono-infected subjects (Table 4) even when adjusting for age and the number of days. Additionally, TGF-β1 was the only immune marker significantly higher in the DENV mono-infected subjects than in the *Plasmodium* mono-infected subjects.

**Table 4:** Matched group comparisons of inflammatory marker levels in subjects with co-infection, dengue virus mono-infection, and *Plasmodium* mono-infection using random effect regression.

Immune marker	Median (Interquartile Range)			
	Co-infection (n = 17)	Dengue virus (n = 51)	*Plasmodium* (n = 44)	p-value*
**Acute phase proteins**				
IL-1β	0.8 (0.3–1.1)	0.7 (0.3–1.0)	1.4 (0.7–2.2)	**<0.001**^#^
IL-6	17.1 (5.8–27.0)	15.6 (7.8–23.3)	50.4 (18.9–188.2)	**<0.001**^¶^^,^ ^#^
TNFα	6.5 (2.6–32.4)	8.6 (4.8–19.7)	11.9 (4.8–24.3)	0.82
IL-8	17.7 (12.7–27.2)	21.7 (13.8–30.1)	19.4 (13.6–32.5)	0.85
**Chemokines and growth factors**				
MCP1/CCL2	176.3 (113.7–294.5)	221.2 (109.3–408.1)	160.0 (55.3–386.2)	0.07
MIP1α/CCL3	292.3 (210.2–400.6)	295.2 (242.2–421.0)	362.3 (248.1–526.3)	**0.043**^¶^^,^ ^#^
G-CSF	18.21 (7.2–31.9)	18.2 (12.3–25.9)	38.0 (21.6–66.3)	0.091^#^
IL-7	6.5 (3.6–8.6)	9.0 (6.5–11.8)	8.2 (5.1–12.6)	**0.04**
**Th1**				
IL-12p40	22.8 (12.9–28.1)	20.9 (15.7–28.5)	34.1 (23.2–53.1)	**0.002**^¶^^,^ ^#^
IFNγ	9.6 (1.0–76.3)	17.1 (7.2–34.5)	56.7 (22.1–128.3)	**0.001**^¶^^,^ ^#^
**Th2**				
IL-4	0.4 (0.1–1.1)	0.8 (0.5–1.4)	2.2 (0.8–3.7)	**<0.001**^¶^^,^ ^#^
IL-13	1.2 (0.3–3.9)	0.9 (0.3–3.1)	3.5 (1.0–8.2)	**0.010**^¶^^,^ ^#^
**Th17**				
IL-17A	21.2 (10.4–40.2)	27.1 (14.8–35.6)	42.3 (21.3–62.8)	**0.008**^¶^^,^ ^#^
**Regulatory cytokines**				
IL-10	18.6 (2.1–66.4)	11.7 (6.2–22.8)	77.8 (18.7–475.4)	**<0.001**^¶^^,^ ^#^
TGFβ1	7.9 (2.3–14.6)	12.6 (8.2–19.6)	7.7 (2.1–10.2)	**0.017**^#^

*Overall comparison across all three groups using a mixed-random effect regression model after adjusting for patient age and number of days with fever

¶ p < 0.05 when comparing co-infected and *Plasmodium* mono-infected groups in the model

# p < 0.05 when comparing *Plasmodium* mono-infected and dengue virus mono-infected groups in the model

## Discussion

This study adds Peru to other South American countries reporting *Plasmodium*/DENV co-infection in the Amazon region. Our results revealed a strikingly similar inflammatory profile between subjects with co-infection and DENV mono-infection, in sharp contrast to the higher levels of immune markers in those with *Plasmodium* mono-infection. On the other hand, most symptoms occurred with similar frequencies between the three groups, although the study was most likely underpowered to detect differences. Because the Ministry of Health’s malaria microscopy samples were incorporated into this febrile surveillance protocol only during certain time periods over the nine-year study, we did not calculate an incidence of *Plasmodium*/DENV co-infection for this timeframe.

In addition to this investigation, only five studies of *Plasmodium*/DENV co-infection have compared co-infected subjects to those with mono-infection [10, 17, 21, 27, 30]. A study from Pakistan looking at 19 clinical features found only one to be different in co-infected patients, jaundice, and this occurred less often in the co-infected group than either *Plasmodium* or DENV mono-infection [30]. A group from India followed 27 co-infected, 102 *Plasmodium* mono-infected, and 340 DENV mono-infected subjects and compared symptoms and routine laboratory values, including liver function tests and hematologic parameters [21]. *P*. *falciparum* was the most prevalent malaria species in this study, although they observed trends similar to ours where *P*. *vivax* predominated. Specifically: 1) similarities between *Plasmodium*/DENV co-infection and DENV mono-infection; 2) differences between *Plasmodium*/DENV co-infection and *Plasmodium* mono-infection; and 3) no apparent worsened disease during infection with both pathogens. Conversely, the largest comparison study of co-infected subjects, which described 104 co-infected patients in French Guiana, showed that hypotension, thrombocytopenia, and anemia were more common in co-infection than in mono-infection with either pathogen alone [17]. Similarly, a study from Brazil showed more bleeding, hepatomegaly, and jaundice in the co-infected group [27]. Furthermore, a study from Pakistan contrasted 26 co-infected subjects with 52 DENV mono-infected subjects and found that hematocrit, hemoglobin, and platelet levels were all lower in the co-infected group although leukopenia was more common in those with DENV mono-infection [10]. However, symptoms were not compared, tests of statistical significance were not performed, and no attempt was made to match or adjust the comparator groups for other covariates. The differences in the findings among these five studies and ours—with three demonstrating and three not demonstrating worse disease in co-infected subjects—may be due to differences in DENV serotypes, *Plasmodium* species, or outcomes evaluated.

While tempting to hypothesize that *Plasmodium*/DENV co-infection would routinely result in more severe outcomes, as seen in the French Guiana study, most co-infection case reports do not describe severe or fatal outcomes (Table 1). The majority of co-infection cases in the literature occurred in regions, similar to ours, with a predominance of *P*. *vivax*, a malaria species less associated with severe outcomes than *P*. *falciparum* [44]. All case reports describing fatal or near-fatal *Plasmodium*/DENV co-infection involved *P*. *falciparum*, including those from West Africa [6], Indonesia [24], East Timor [9], Pakistan [7], and India [16]. Nevertheless, severe manifestations are still possible when *Plasmodium*/DENV co-infection occurs with *P*. *vivax*, as demonstrated in the larger studies from French Guiana and Brazil [17, 27].

Both malaria and dengue have been associated with a strong activation of acute phase response (IL-6, TNF-α, and IL1-β) and Th1 cytokines (IFN-γ and IL-12). Therefore, we hypothesized that co-infection may result in synergistic activation of these pathways and overall higher levels of pro-inflammatory immune markers in the serum. However, our comprehensive characterization of multiple cytokines, chemokines, and growth factors using a highly sensitive multiplex platform revealed that *Plasmodium* mono-infected subjects had higher levels of many of the immune markers evaluated compared to DENV mono-infected subjects even after matching and adjusting for other covariates. The lower levels of inflammation in the co-infected group were, in fact, similar to DENV mono-infected subjects. Consistent with our results, *P*. *falciparum* infection has been associated with a greater activation of inflammatory response compared with DENV infection, including higher levels of IFN-γ and IL-10 [45] and, in some cases, *P*. *vivax* may have an even more robust inflammatory response than *P*. *falciparum* [46].

The regulatory marker TGF-β1 was the only immune marker with higher levels in DENV mono-infected individuals compared to *Plasmodium* mono-infected and co-infected subjects. Prior studies of TGF-β1 in DENV mono-infection have demonstrated a positive correlation with symptomatic disease. A group from French Polynesia showed that TGF-β1 levels were highest in those with dengue hemorrhagic fever versus dengue fever; likewise, higher levels were found in those with dengue fever than healthy controls [47]. A study of subjects with dengue in India similarly showed a correlation of TGF-β1 levels with severity and also length of disease [48]. In our study and others, TGF-β1 levels in malaria show an opposite trend compared with dengue. TGF-β1 levels were higher in healthy Tanzanian controls compared to those with *P*. *falciparum* malaria. In the same study, TGF-β1 levels were inversely correlated with malaria severity [49]. Parasite density in children from Gabon with *P*. *falciparum* malaria was also shown to be inversely related to TGF-β1 levels and highest in those without malaria [50]. Our findings are consistent with these four studies, which together demonstrate high TGF-β1 levels in symptomatic dengue and low TGF-β1 levels in symptomatic malaria.

Following infection with DENV, viremia first peaks and then becomes undetectable over a timespan usually lasting less than three weeks. Chronic infection is not a characteristic of dengue. Asymptomatic, long-lasting *Plasmodium* infections, however, are common both in *P*. *vivax* and *P*. *falciparum* malaria [51]. In the case of *P*. *vivax*, a clinically dormant hypnozoite form exists that may reactivate months or years later during a physiological stressor, such as DENV infection. The scenario of acute, symptomatic DENV infection occurring in a person with clinically silent *Plasmodium* infection would explain why the immune marker profile of our co-infected subjects nearly always resembled DENV mono-infected subjects. In contrast, our subjects with *Plasmodium* mono-infection most likely presented with fever directly attributable to malaria and therefore displayed a vastly different immune marker response from the other two groups. This same reasoning would account for why *Plasmodium*/DENV co-infection did not result in a higher frequency of symptoms than mono-infection with DENV. Further studies in humans or animal models are needed to better understand the effect of DENV infection on the course of *Plasmodium* infection or vice versa.

A few caveats are worth noting. First, given the retrospective nature of our study, we measured soluble immune marker levels in the serum which are not pathogen-specific, so it is possible that other infections or underlying medical conditions may have confounded our findings. However, we individually matched mono-infected subjects to every co-infected subject in terms of gender, enrollment site, and period (±3 months). In addition, we performed immune marker testing on all samples at the same time in order to avoid batch variability. Second, due to the design of the studies, we did not collect clinical laboratory data, such as platelet levels, creatinine, liver enzymes, or hematocrit, which can be used to stratify disease severity and has been utilized to differentiate between *Plasmodium* and DENV mono-infection in other studies [52]. Furthermore, we only assessed our subjects at one point in time, which could have missed evolution to a more severe disease state, such as dengue hemorrhagic fever, which classically follows an initial, less severe dengue fever stage. However, dengue hemorrhagic fever was virtually non-existent in Iquitos and Puerto Maldonado during the time of our study. Next, our study occurred during a timeframe when DENV-3 and *P*. *vivax* were prominent in the study regions and infection with *P*. *falciparum* was not associated with severe outcomes, so our results may not be generalizable to areas in the world with different DENV serotypes or a predominance of *P*. *falciparum* malaria. Finally, our sample size may have been too small to detect differences in most symptoms; however, large and statistically significant differences in immune marker levels were still able to be observed, even though the study may have been underpowered to detect more nuanced differences.

Our results verify the existence of *Plasmodium*/DENV co-infection in the Amazon region of Peru. Therefore, diagnosis of one disease in a febrile patient in this area does not preclude infection with the other. This realization is significant because malaria can be treated with prompt administration of anti-malarial medications, which could be mistakenly withheld if only DENV were presumed in a co-infected patient. Although effective anti-viral treatment is not available for dengue disease, early identification is crucial for the supportive management of severe cases [53]. For the time period of our study, co-infection did not yield more symptoms or higher immune marker levels than mono-infection alone. While this is momentarily reassuring, the recent discovery of more life-threatening forms of malaria [54] and DENV [55] in the region emphasize the future importance of not overlooking either of these potentially fatal but treatable diseases.

## Supporting Information

S1 Checklist STROBE Checklist. (DOC)
